# Secondary effects of dialectical behaviour therapy on social functioning, quality of life, and autism traits in autistic adults with suicidality

**DOI:** 10.1177/13623613241302875

**Published:** 2024-12-14

**Authors:** Anne Huntjens, LMC (Wies) van den Bosch, Bram Sizoo, Ad Kerkhof, Filip Smit, Mark van der Gaag

**Affiliations:** 1 Vrije Universiteit Amsterdam, The Netherlands; 2 Parnassia Psychiatric Institute, The Netherlands; 3 Dialexis, The Netherlands; 4 University of Amsterdam, The Netherlands

**Keywords:** autism, autism traits, dialectical behaviour therapy, quality of life, social functioning, suicidal behaviour

## Abstract

The effect of psychological treatment on social functioning, quality of life and autism traits in autistic people with suicidal behaviour remains largely unknown. At six Dutch mental health centres, 123 adult outpatients with *Diagnostic and Statistical Manual of Mental Disorders* (5th edition) diagnosed autism spectrum condition and suicidal behaviours were randomly assigned to dialectical behaviour therapy (*n* = 63) or treatment as usual (*n* = 60) to address their suicidal behaviours. This article analysed secondary outcomes on social functioning, quality of life and autism traits. Outcomes were compared at baseline, post-treatment at 6 months, and 12-month follow-up. At post-treatment, both social functioning (*p* < 0.001) and quality of life (*p* = 0.002) were significantly improved in the treatment condition compared to the control condition and remained so at 12-month follow-up (*p* = 0.003; *p* = 0.002). Autism traits did not differ between conditions. Autistic individuals with suicidal behaviours benefit modestly from treatment with dialectical behaviour therapy in social functioning and quality of life.

**Lay abstract:**

Dialectical behaviour therapy is a comprehensive treatment that helps individuals improve distress tolerance, mindfulness, interpersonal effectiveness and emotion regulation. It is commonly used to assist those experiencing self-harm and suicidal thoughts or behaviours. Despite its broad application, our understanding of how dialectical behaviour therapy impacts autistic individuals with suicidal behaviour remains limited. This study compared dialectical behaviour therapy with treatment as usual in 123 autistic adults experiencing suicidal behaviours. Participants were recruited from six mental health centres, with 63 receiving dialectical behaviour therapy and 60 receiving treatment as usual. The study assessed outcomes such as social functioning, quality of life and specific autism traits over 12 months. Findings revealed that dialectical behaviour therapy led to significant improvements in social functioning and quality of life compared to treatment as usual, though there were no effects on autism traits. These improvements suggest that dialectical behaviour therapy holds promise as an effective treatment for autistic individuals grappling with suicidal behaviour. The findings strongly support the broader implementation of dialectical behaviour therapy in mental health centres, especially given the limited treatment options available for autistic individuals with suicidal tendencies.

## Introduction

Autism spectrum condition is a lifelong neurodevelopmental condition characterised by social communication, interaction differences and restricted, repetitive patterns of behaviour, interests or activities (American Psychiatric Association, 2013). Previous narrative reviews have consistently demonstrated an increased risk of suicidal behaviour among autistic adults (Hedley & Uljarević, 2018; Zahid & Upthegrove, 2017). A recent meta-analysis found a 12-month prevalence of 25% for suicidal ideation and 14% for suicide attempts (Huntjens et al., 2024a).

Among the risk factors for suicidality in autistic individuals are depression, poor social functioning (Hedley et al., 2018), likely lower quality of life and possibly the intensity of autism traits (Cassidy et al., 2018). Depression, in general, and specific characteristics such as the inability to imagine alternative strategies (South et al., 2020), rumination, low self-esteem (Arwert & Sizoo, 2020) and feelings of defeat and entrapment (O’Connor & Kirtley, 2018), significantly contribute to suicidality in autism.

Challenges in social functioning, including social exclusion and isolation, are also notable risk factors for suicidality (Camm-Crosbie et al., 2019; Cassidy, 2020; Cassidy et al., 2018; Crane et al., 2019; Hedley & Uljarević, 2018; Mitchell et al., 2021). Feelings of thwarted belonging (Pelton et al., 2020) and lack of societal acceptance (Cage et al., 2018) further contribute to the high rates of suicidal behaviour among autistic individuals, as well as those with high autistic traits (Richards et al., 2019).

Autistic adults frequently report a lower quality of life compared to the general population, as demonstrated in numerous studies (Jennes-Coussens et al., 2006; Kamio et al., 2013; Lawson et al., 2020; Mason et al., 2018; McConachie et al., 2018; Oakley et al., 2021; van Heijst & Geurts, 2015).

The intensity of autism traits, such as social communication challenges, is associated with a lower subjective quality of life among many autistic individuals (Chiang & Wineman, 2014; Pisula et al., 2015; Steensel & Bögel, 2012). However, the impact varies widely among individuals, influenced by personal differences, life stages, comorbid conditions, environmental factors, life events and support (Oakley et al., 2021).

Recently, we evaluated the effectiveness of dialectical behaviour therapy (DBT) in treating suicidal behaviour in autistic adults through a multicentre, assessor-blind controlled trial at six Dutch mental health centres involving 123 adult outpatients (Huntjens et al., 2024b). Participants were randomly assigned to DBT or treatment as usual (TAU), with assessments at baseline, end of treatment at 6 months and follow-up at 12 months. The findings were that DBT not only reduced suicidal ideation and suicide attempts but also mitigated symptoms of depression (Huntjens et al., 2024b).

While DBT has traditionally been studied in individuals with personality disorders, demonstrating improvements in well-being and quality of life (Robinson et al., 2018; Webb et al., 2023), it has also been shown to enhance social functioning (Carter et al., 2010; McMain et al., 2022; Webb et al., 2023). DBT addresses multiple aspects of social interaction by incorporating individual therapy, skills training to enhance and strengthen social skills, a therapist consultation team and phone consultations as needed (Linehan, 1993). The DBT skills group includes modules on mindfulness, distress tolerance, emotion regulation and interpersonal effectiveness, conducted in a classroom format with weekly practice and homework assignments.

This study analyses secondary outcome data from a randomised controlled trial (RCT) targeting suicidal behaviour in autistic individuals. We hypothesise that DBT will improve social functioning and quality of life in autistic people as it has for those with personality disorders. In addition, autism traits are associated with quality of life. Although no research has documented the effect of DBT on autism traits, this study will explore the effects of DBT on these traits.

## Methods

This study involves a secondary data analysis of data collected from the original RCT titled “The effectiveness and safety of dialectical behaviour therapy for suicidal ideation and suicidal behaviour in autistic adults’ (Huntjens et al., 2024b). The secondary analysis utilises data from the Personal and Social Performance Scale (PSP) for social functioning (Morosini et al., 2000), the Manchester Short Assessment of Quality of Life (MANSA) for quality of life (Priebe et al., 1999) and autism traits, which were quantitatively measured using the Social Responsiveness Scale-Adult version (SRS-A) (Chan et al., 2017; Noens et al., 2012) collected during the original trial.

### Design

This study is a single-blind RCT with two parallel arms (DBT and TAU) and measurements at baseline (t_0_), at the end of DBT treatment after 6 months (t_6_) and at a follow-up at 12 months (t_12_). To increase clinical relevance, the trial was conducted across six mental health services with minimal exclusion criteria. With *N* = 128 consenting participants (64 in each arm), the study would be well-powered to detect a medium-sized standardised effect of *d* = 0.50 in a test with a power of (1−β) ⩾ 0.80 and α ⩽ 0.05 (two-tailed). The study was registered at Current Controlled Trials (ISRCTN 96632579) and approved by the Medical Ethics Committee of the VU University Medical Centre (NL59497.029.17) in March 2018. Further details are available from the study protocol (Huntjens et al., 2020).

### Participants

Participants were recruited from six specialised autism departments within mental health services in the Netherlands. Prior to inclusion, all participants had received an autism diagnosis from experienced clinicians following national guidelines (Kan et al., 2013). All patients were already in treatment and were informed about the study by their treating therapist. The participants were screened using the autism-spectrum quotient (AQ-short; Hoekstra et al., 2011) to verify their autism diagnosis and with the Suicidal Ideation Attributes Scale (SIDAS; van Spijker et al., 2014) and Lifetime Parasuicide Count (LPC; Comtois & Linehan, 1999) to assess inclusion criteria. Written informed consent was obtained from patients who were eligible and willing to participate. The inclusion criteria were (1) age 18 to 65 years, (2) fulfilment of Diagnostic and Statistical Manual of Mental Disorders (5th edition; *DSM*-V) criteria for autism spectrum condition (American Psychiatric Association, 2013), (3) outpatient status, (4) suicidal ideation defined as SIDAS score ⩾ 21 (van Spijker et al., 2014) and (5) level of suicidal behaviour rated as severe on the LPC (score of 2 on each item; Comtois & Linehan, 1999). Exclusion criteria were (1) IQ < 80 assessed with Wechsler Adult Intelligence Scale–Fourth Edition (WAIS-IV) only if baseline testing was difficult due to intellectual deficits (Wechsler, 2008), (2) addiction to drugs and need for clinical detoxification and (3) insufficient mastery of the Dutch language.

### Measures

Social functioning was assessed using the PSP (Morosini et al., 2000). The PSP is a clinician-rated instrument designed to distinguish between psychopathological and psychosocial aspects, providing a more precise operationalisation of occupational, socialand personal functioning domains. It evaluates social functioning across four primary domains: (1) socially useful activities (e.g. housekeeping, voluntary work) including work and study; (2) personal and social relationships (e.g. partner, family and friends); (3) self-care (e.g. personal hygiene, care of one’s appearance); and (4) disruptive and aggressive behaviour. Each domain’s impairment is rated on a six-point scale: absent (0), mild (1), moderate (2), marked (3), severe (4) and very severe (5). A higher score within a domain indicates worse functioning in that particular area. The PSP total score, ranging from 1 to 100, is not merely a sum of the individual domain scores. Instead, it is calculated through a multiple-weighting process that integrates the four domains of functioning. For instance, a total score of 41–50 may indicate significant impairments in work and social relationships, work and self-care, social relationships and self-care or serious dysfunction in a single domain, even in the absence of aggressive behaviour. By combining and weighting nominal categories, a numerical score is generated (see Table 1 in the Supplemental File).

**Table 1.** Baseline characteristics of participants.

	DBT (*n* = 63)	TAU (*n* = 60)	All (*n* = 123)
Age in years, *M* (*SD*)	36.9 (10.6)	37.9 (12.1)	37.4 (11.3)
Gender, *n* (%)			
Male	33 (52)	31 (52)	64 (52)
Female	30 (48)	28 (47)	58 (47)
Non-binary	0 (0)	1 (2)	1 (1)
Non-Dutch origin, *n* (%)	2 (3)	1 (2)	3 (2)
Living conditions, *n* (%)			
Married or cohabitating	20 (32)	15 (25)	35 (28)
Children	18 (29)	11 (18)	29 (24)
Alone	24 (38)	30 (50)	54 (44)
Sheltered housing	6 (10)	7 (12)	13 (11)
Employed, *n* (%)	20 (32)	23 (38)	43 (35)
Education, *n* (%)			
University	8 (13)	6 (10)	14 (11)
Higher professional	4 (6)	8 (13)	12 (10)
Middle vocational	22 (35)	19 (32)	41 (33)
Lower vocational	2 (3)	0 (0)	2 (2)
Secondary	21 (35)	22 (37)	43 (35)
*DSM*-V diagnosis			
ASD, *n* (%)	63 (100)	60 (100)	123 (100)
Age at diagnosis of ASD, *M* (*SD*)	29.2 (12.7)	30.5 (14.3)	29.8 (13.4)
Number of diagnoses, *M* (*SD*)	2.0 (0.7)	1.8 (0.7)	1.9 (0.7)
Prior *DSM* diagnoses^a^ (%)	54 (86)	46 (77)	81 (100)
*DSM* comorbidity			
Depressive disorder *n* (%)	28 (44)	20 (33)	48 (39)
Personality disorder *n* (%)	8 (10)	4 (7)	12 (10)
Post-traumatic stress disorder	5 (8)	4 (7)	9 (7)
Anxiety disorder *n* (%)	2 (3)	4 (7)	6 (5)
Obsessive compulsive disorder *n* (%)	4 (6)	3 (5)	7 (6)
Attention deficit hyperactivity disorder	14 (22)	15 (25)	29 (24)
Bipolar disorder *n* (%)	1 (2)	–	1 (1)
Eating disorder *n* (%)	4 (6)	4 (7)	8 (7)
Substance-related disorder *n* (%)	7 (11)	4 (7)	11 (9)
Medication use^b^, *n* (%)			
Antidepressants	36 (57)	28 (47)	64 (52)
Antipsychotics	23 (37)	21 (35)	44 (36)
ADHD	10 (16)	9 (15)	19 (15)
Outcomes variables, *M* (*SD*)			
Social functioning (PSP)	39.9 (12.0)	40.8 (13.2)	40.3 (11.7)
Quality of life (MANSA)	3.37 (0.96)	3.48 (0.92)	3.39 (0.81)
Autism traits, (SRS-A)	89.3 (20.4)	93.3 (17.5)	91.5 (16.9)

DBT: dialectical behaviour therapy; TAU: treatment as usual; *n*: number of participants; M: mean; *SD*: standard deviation; *DSM*-V: Diagnostic and Statistical Manual of Mental Disorders, Fifth Edition; ASD: autism spectrum disorder; ADHD: attention deficit hyperactivity disorder.

a Prior *DSM* diagnoses are available on request.

b Which (and dosage) antidepressants, antipsychotics and ADHD medication are available on request.

The total score varies from 1 to 100 and is divided into 10 equal intervals, where a change of at least 10 points is considered clinically meaningful (Lee et al., 2016). Scores from 100 to 70 indicate mild difficulties, 70 to 31 manifest disabilities of varying degrees and below 30 points denote severe impairment requiring intensive support or supervision (Morosini et al., 2000). The PSP scale was selected for its multidimensional nature, robust psychometric and minimal influence from specific patient symptoms (Fernández-Lafitte et al., 2022). While not specifically validated for autistic individuals, the PSP scale may be suitable due to its broad applicability, holistic approach, objective clinician ratings, comparability with other studies, focus on daily functioning and relevance to key functional domains affected by autism. To gain a comprehensive understanding of participants’ areas of difficulty and improvement, we initially assessed the total score followed by individual domain scores. In addition, the PSP scale demonstrated strong reliability in our sample (Cronbach’s alpha = 0.87).

Quality of life was assessed using the MANSA (Priebe et al., 1999), designed to evaluate various facets of life satisfaction. It comprises four objective questions and 12 subjective questions that assess overall satisfaction with life in domains such as employment status, financial situation, friendships, leisure activities, living conditions, personal safety, household composition, intimacy, familial relationships, physical health and mental well-being. Responses are rated on a 7-point Likert-type scale, ranging from 1 (Could not be worse) to 7 (Could not be better). In addition, four objective questions require binary responses (‘yes’ or ‘no’). This study employed an authorised Dutch translation of the MANSA (Nieuwenhuizen et al., 2000; van Nieuwenhuizen et al., 1998). The MANSA provides two scoring methods: aggregating scores from its 12 subjective items or calculating the mean score of these items (Nieuwenhuizen et al., 2000). This study employed the summed scores method, where a higher MANSA sum score indicates a higher perceived quality of life. The MANSA was chosen for its robust psychometric properties, including high correlations with other quality-of-life measures (Björkman & Svensson, 2005; Priebe et al., 1999), sensitivity to longitudinal changes (Priebe et al., 2011; Slade et al., 2006) and demonstrated reliability (Cronbach’s alpha = 0.78) in our sample. Moreover, MANSA scores have shown independence from the type of treatment setting (Dehn et al., 2022; Priebe et al., 2011) further supporting its suitability despite the absence of specific validation for autistic individuals (Priebe et al., 1999).

Autism traits were quantitatively measured using the SRS-A (Chan et al., 2017; Noens et al., 2012). This self-report questionnaire has four dimensions of autism: ‘social awareness’ measures the ability to perceive social cues (e.g. ‘Is aware of what others are thinking or feeling’); ‘social communication’ evaluates expressive social communication (e.g. ‘Avoids eye contact or has unusual eye contact’); ‘social motivation’ gauges the general motivation to engage in social-interpersonal behaviour, including aspects of social anxiety, inhibition and empathic orientation (e.g. ‘Would rather be alone than with others’); and ‘restricted interests and repetitive behaviour’, which measure stereotypical behaviours or highly restricted interests (e.g. ‘Has an unusually narrow range of interests’). The SRS-A consists of 64 items rated on a Likert-type scale from 1 ‘not true’ to 4 ‘almost always true’. A higher total score indicated more autism traits. The SRS-A has demonstrated excellent psychometric properties, and it was chosen for its focus on deficits aligning with the social domain of autism (Noens et al., 2012). In our study, we examined the overall outcomes of the SRS-A, and for a more nuanced understanding, we differentiated between social and behavioural traits.

### Procedure

The demographic characteristics of the participants were recorded at baseline. Assessments were assessed at baseline, post-treatment at 6 months and follow-up at 12 months. After a baseline assessment, 123 patients were randomly assigned to either DBT or TAU. The independent randomisation office of the Parnassia Psychiatric Institute randomised participants using www.randomizer.org. Research assistants blinded to treatment allocation performed the post-treatment and follow-up assessments. After the 12-month follow-up, participants in the TAU condition were offered DBT treatment. All participants received financial compensation of €25 (USD 27) for each assessment.

### Intervention

The intervention was based on the comprehensive DBT framework, which included: (1) weekly individual cognitive-behavioural psychotherapy sessions lasting 45 min with the primary therapist, (2) weekly 2 h and 15 min skills training group, (3) provision of telephone coaching with their individual therapist if necessary and (4) weekly 1 h therapist consultation (Linehan, 1993). Participants assigned to the DBT group underwent pretreatment, which involved an introduction to the therapist and familiarisation with the demands and expectations of DBT treatment. The treatment period was shortened from 1 year to 6 months by increasing the frequency of skills training sessions from once to twice a week. This adjustment provided more opportunities for feedback, rehearsal, practice and enhanced skill learning, customising the training to meet the specific needs of autistic adults (Anderson & Morris, 2006; Geurts et al., 2009; Hartmann et al., 2012). The 26-week skill training in this study was adapted from the study by Neacsiu et al. (2014). Prior to the study, some textual modifications were made to the DBT manual in collaboration with autistic adults to improve clarity and comprehension. Specifically, explanations of certain skills were simplified without changing the content, making the text more readable. In addition, mindfulness exercises introduced at the beginning of skill training were explained with precise and straightforward instructions tailored to the unique needs of autistic individuals (Spek et al., 2013). When a skill training was missed, the participants were asked to catch up by watching video recordings made of all the training sessions. The therapists kept track of self-reported viewing by the participants. Participants who missed four sessions in a row were considered treatment dropouts. All therapists in the DBT condition were psychologists or psychiatrists, and skills trainers were psychologists, registered nurses or social workers (*N* = 36).

The control group received treatment as usual. This was any form of treatment for suicidal behaviour in autism that is common within the Dutch mental health system and was given in weekly 45-min sessions with a psychotherapist or social worker. Any form and intensity of treatment were permitted to ensure that the TAU condition realistically mirrored current clinical practice intensity, for example, psycho-education, social skills training, emotion regulation therapy, cognitive-behavioural therapy, trauma therapy, delivered in both group and individual formats, either independently or concurrently. Participants who dropped out of treatment were asked to complete the post-treatment and follow-up assessments.

### Statistical analysis

Analyses were performed with the statistical software Stata (version 17.0) and SPSS (version 27). The analyses were based on the intention-to-treat (ITT) principle, thus including all participants as randomised. ITT analysis was achieved using mixed modelling. The Gaussian-distributed PSP, MANSA and SRS-A scales were regressed on Condition (0/1), Time and Time × Condition interaction with baseline scores on the outcome variables as a covariate for baseline adjustment. This was done in the fixed part of the equation. Participant ID was modelled as a level in the random part of the equation. An unstructured between-group variance and covariance structure was assumed. It was an a priori decision not to include the six treatment sites as an extra level, in that there were only six sites. Instead, we modelled the nesting of patients within sites by a series of site (0/1) indicators in the fixed part of the equation.

### Sensitivity analyses

It was an a priori decision to assess the robustness of the base case analyses in a sensitivity analysis using last-observation-carried-forward (LOCF) imputation of missing observations caused by study dropout. To that end, all mixed models were repeated after LOCF imputation.

In response to the challenges posed by the COVID-19 pandemic, a post hoc decision was made to conduct an additional sensitivity analysis, examining the impact of disrupted access to healthcare and the transitions to telehealth during three COVID-19 lockdowns (Rijksoverheid, 2021). The swift transition from face-to-face treatment to telehealth, primarily using Zoom, was prompted by national lockdowns and social distancing regulations (Huntjens et al., 2024b). Proactively addressing challenges, the study facilitated telehealth through Zoom and overcame obstacles such as patients lacking smartphones by providing borrowed devices. Assessments, usually conducted face-to-face, were shifted to video calls during lockdowns. Three COVID lockdown indicators were created for each participant at each data collection wave (baseline, post-treatment and follow-up). These indicators were set at 1 if the participant’s access to face-to-face care was disrupted by a lockdown at that time and 0 otherwise. The COVID lockdown indicators were included as time-varying confounders in the mixed model equations. Subsequently, the base case analyses were compared with the lockdown-adjusted analyses.

### Community involvement

The study was conducted with autistic adults and therapists from routine mental health services. In collaboration with autistic adults, text modifications were made to the DBT manual before the study’s commencement, simplifying explanations of certain DBT skills to make them more concrete and understandable for autistic individuals. The first author received significant language feedback from many participants during the trial. This feedback was utilised after the study to improve the treatment material and ultimately incorporated into the DBT manual. The study included disseminating 10 newsletters to all participants and therapists, providing up-to-date study information, and conducting interviews with the participants and therapists to gather their feedback and experiences. Once this study has been published, participants will be informed, and details of the results will be sent in a study newsletter suitable for a non-specialist audience.

## Results

### Sample at baseline

**Figure 1.**
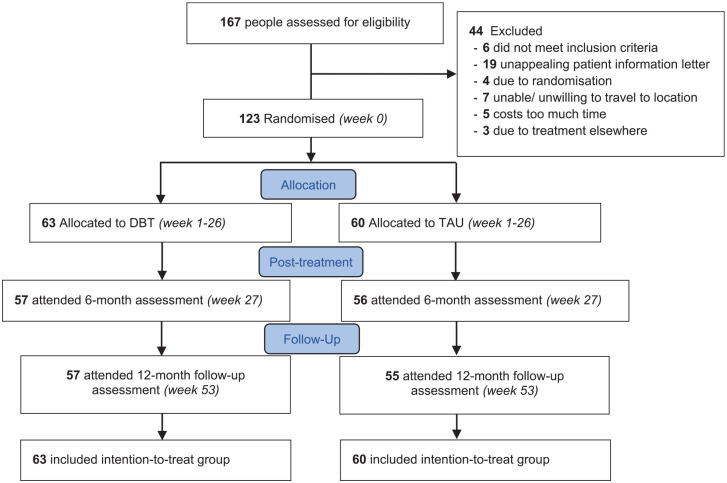
Flow of participants. DBT: dialectical behaviour therapy; TAU: treatment as usual; PSP: personal and social functioning; MANSA: the Manchester short assessment of quality of life; SRS-A: Social Responsiveness Scale.

Recruitment was from September 2018 to December 2022, with 167 potential participants assessed for eligibility. Of these, 44 did not meet inclusion criteria and 123 participants were randomly assigned to either the DBT group (*n* = 63) or the TAU group (*n* = 60; see Figure 1). Table 1 shows the baseline characteristics of the secondary symptoms per condition and in total. No baseline differences were observed between the conditions on any variables. Similarly, no significant differences were noted between patients with and without medication changes at baseline, after treatment or at follow-up (data not shown).

**Figure 2.**
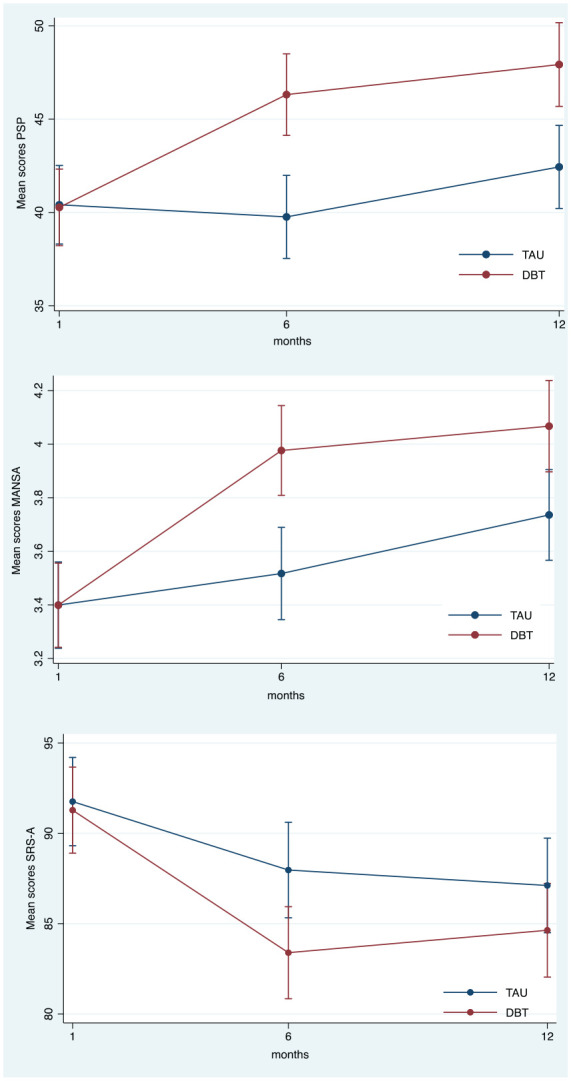
Change in secondary outcomes by condition over time. DBT: dialectical behaviour therapy; TAU: treatment as usual; PSP: personal and social functioning; MANSA: the Manchester short assessment of quality of life; SRS-A: Social Responsiveness Scale.

Social functioning (PSP) improved significantly more with DBT than TAU at post-treatment as well as at the 12-month follow-up (see Table 2 and Figure 2). For the four domains of the PSP scale, the nominal categories were assigned scores from 0 to 5. The results showed improvement across all domains for DBT; however, statistical significance was only reached in self-care at the 12-month follow-up. No significant improvements were observed in the work, social relations or aggressive domains, either post-treatment or at follow-up, see Table 2.

**Table 2.** Means, standard deviations and test results.

	DBT			TAU			Time^a^					Time × treatment^b^		
	t_0_	t_6_	t_12_	t_0_	t_6_	t_12_	t_6_	*p*	t_12_	*p*	DBT × t_6_	*p*	DBT × t_12_	*p*
	*n* = 63	*n* = 57	*n* = 57	*n* = 60	*n* = 56	*n* = 55								
	*M* (*SD*)	*M* (*SD*)	*M* (*SD*)	*M* (*SD*)	*M* (*SD*)	*M* (*SD*)	*b* (95% CI)		*b* (95% CI)		*b* (95% CI)		*b* (95% CI)	
PSP	39.9 (12.0)	46.0 (13.3)	47.5 (14.9)	40.8 (13.2)	41.1 (14.8)	42.9 (14.7)	–0.53 (–3.15 to 2.09)	0.691	1.93 (–0.68 to 4.56)	0.148	6.69 (3.01–10.3)	**0.000**	5.62 (1.91–9.33)	**0.003**
PSP_work	2.98	2.88	2.53	3.11	3.03	2.67	–0.57 (–0.35 to 0.19)	0.572	–3.54 (–0.77 to –0.22)	**0.000**	–0.47 (–0.47 to 0.29)	0.641	0.92 (–0.36 to 0.40)	0.929
PSP_rel	2.57	2.14	1.83	2.55	2.26	2.05	–2.81 (–0.52 to –0.94)	**0.005**	–5.05 (–0.77 to –0.34)	**0.000**	–0.91 (–0.44 to 0.16)	0.365	–1.14 (–0.48 to 0.12)	0.254
PSP_care	2.55	2.16	2.03	2.44	2.42	2.03	–0.14 (–0.26 to 0.23)	0.888	–0.63 (–0.32 to 0.16)	0.531	–1.79 (–0.66 to –0.03)	0.073	–2.13 (–0.73 to –0.02)	**0.033**
PSP_agres	1.46	.096	1.15	1.46	1.32	1.11	–0.56 (–0.43 to 0.24)	0.575	–1.45 (–0.56 to 0.87)	0.414	–1.50 (–0.83 to 0.11)	0.135	–0.29 (–0.54 to 0.40)	0.773
MANSA	3.37 (0.96)	3.91 (1.07)	4.02 (1.13)	3.48 (0.92)	3.66 (0.90)	3.87 (1.04)	0.10 (–0.10 to 0.31)	0.319	0.31 (0.11–0.52)	**0.002**	0.45 (0.17–0.74)	**0.002**	0.31 (0.04–0.61)	0.024
SRS-A	89.3 (20.4)	81.6 (15.9)	82.9 (21.3)	93.3 (17.5)	90.6 (19.1)	88.9 (18.2)	–3.36 (–6.64 to –0.86)	0.044	–4.47 (–7.73 to 1.21)	**0.007**	–4.10 (–8.66 to 0.46)	0.078	–2.00 (–6.58 to 2.57)	0.390

DBT: dialectical behaviour therapy; TAU: treatment as usual; t_0_: baseline; t_6_: post-treatment; t_12_: 12-month follow-up assessment; *M*: mean; *SD*: standard deviation; *b*: regression coefficient; CI: confidence interval; *n*: number of participants; PSP: Personal and Social Performance Scale; PSP_work: PSP work domain; PSP_rel: PSP relation domain; PSP_care: PSP care domain; PSP_agres: PSP agressive domain; MANSA: Manchester short assessment of quality of life; SRS-A: Social Responsiveness Scale-adult version.

a Values indicate the *p*-values of time of the multilevel models; *p*-values < 0.05 are presented in bold.

b Values indicate the *p*-values of time × treatment of the multilevel models; *p*-values < 0.05 are presented in bold.

Quality of life (MANSA) improved significantly more in DBT than in TAU at post-treatment. At follow-up, these treatment effects remained significant and in favour of DBT, see Table 2. Figure 2 illustrates these treatment effects by condition and over time.

However, for traits of autism (SRS-A), we observed no statistically significant effects.

### Sensitivity analyses

To ascertain the robustness of the main analyses conducted with the mixed models, a pre-planned sensitivity analysis was conducted in which missing observations due to dropout were imputed using the LOCF method. Table 3 compares regression coefficients, standard errors and *p*-values for time effects and condition × time (DBT vs TAU) from base case analyses, LOCF sensitivity analysis, and COVID lockdown-adjusted analyses for PSP social functioning, MANSA quality of life and SRS-A autism traits. Overall, minimal differences were noted between the main and LOCF sensitivity analyses.

**Table 3.** Main vs Sensitivity vs Covid over time.

Outcome analysis	Time (month)		Time × treatment (month)		Time (month)		Time × treatment (month)		Time (month)		Time × treatment (month)	
	6	12	6	12	6	12	6	12	6	12	6	12
PSP												
Base Case	–0.5330	193.7	6.692	5.626	1.339	1.339	1.876	1.892	0.691	0.148	**0.000**	**0.003**
Sens LOCF	–0.5166	1.6	5.897	5.019	1.239	1.239	1.731	1.731	0.677	0.197	**0.001**	**0.004**
Sens COVID	–0.7334	0.248	5.895	4.984	1.593	1.651	1.728	1.728	0.645	0.881	**0.001**	**0.004**
MANSA												
Base Case	0.1045	0.3189	0.4595	0.3317	0.1050	0.1037	0.1495	0.1465	0.319	**0.002**	**0.002**	**0.024**
Sens LOCF	0.0818	0.2969	0.4131	0.2860	0.0968	0.0968	0.1335	0.1335	0.398	**0.002**	**0.002**	**0.035**
Sens COVID	0.0715	0.1369	0.4125	0.2820	0.1240	0.1284	0.1346	0.1346	0.564	0.286	**0.002**	**0.036**
SRS-A												
Base Case	–3.368	–4.471	–4.100	–2.007	1.674	1.664	2.330	2.335	**0.044**	**0.007**	0.078	0.390
Sens LOCF	–2.883	–4.45	–3.45	–0.7722	1.525	1.525	2.131	2.131	0.059	**0.004**	0.106	0.717
Sens COVID	–1.278	–2.935	–3.429	–0.7382	1.942	2.010	2.112	2.112	0.510	0.144	0.105	0.727

PSP: Personal and Social Performance Scale; Base Case: base case analysis; Sens: sensitivity analysis; LOCF: last observation carried forward; Sens COVID: lockdown adjusted analyses; MANSA: Manchester short assessment of quality of life; SRS-A: Social Responsiveness Scale-Adult version.

Statistically significant values at *p* < 0.05 are presented in bold.

Regarding the comparison between COVID-adjusted and main analyses, slight differences were found in *b*-coefficients favouring DBT, with negligible changes in *p*-values for PSP, see Table 3. However, DBT lost significance at post-treatment and follow-up in SRS-A and MANSA adjusted analyses. Time effects on SRS-A at post-treatment and follow-up and MANSA at follow-up lost significance in adjusted analyses. In summary, lockdown-adjusted analyses did not materially alter the original conclusions.

## Discussion

### Key findings

This study focused on examining the secondary effects of DBT on social functioning, quality of life and autism traits in autistic individuals with suicidal behaviour. While the larger RCT established the efficacy of DBT in reducing suicidal ideation and attempts (Huntjens et al., 2024b), this study specifically explores how these secondary outcomes are impacted by the treatment. The results are consistent with our hypotheses that social functioning and quality of life significantly improved in the DBT group compared to the TAU group, both at 6 and at 12 months follow-up. Autism traits showed no group differences.

### Findings in context

The literature review presented in the introduction highlights the significant challenges in addressing suicidal behaviour among autistic individuals, emphasising the scarcity of research on effective interventions such as DBT and other psychological treatments. Factors such as diminished well-being and limited social engagement are recognised as potentially exacerbating the severity of suicidal behaviour in autistic individuals (Camm-Crosbie et al., 2019; Cassidy, 2020; Cassidy et al., 2018; Crane et al., 2019; Hedley & Uljarević, 2018; Mitchell et al., 2021). Emerging research suggests that reductions in suicidality coincide with improvements in social functioning and overall quality of life ((Bemmouna et al., 2022; Carter et al., 2010; McMain et al., 2022). Expanding on these findings, this study explores significant enhancements in social functioning and quality of life observed in the DBT group, within a larger RCT investigating the effectiveness of DBT for suicidal behaviours in autistic adults (Huntjens et al., 2024b). It must be emphasised that the improvements in social functioning and quality of life are only modest. The change in PSP scores is approximately 6 points. While this is statistically significant, it does not meet the threshold of 10 points required for clinical significance (Lee et al., 2016). The separate domains generally show non-significant improvements. The statistical significance observed in self-care is likely due to a slight improvement in the DBT condition, coupled with a slight worsening in the TAU condition.

Improvement in social functioning and quality of life was also observed in the TAU group. This improvement may possibly reflect a regression-to-the-mean effect, considering that participants who were initially at high risk for suicide might naturally present with deteriorated social functioning and quality of life prior to treatment. In addition, the enhanced outcomes observed at post-treatment and follow-up in the TAU group might also reflect the beneficial influence of therapeutic support and mental health services (Camm-Crosbie et al., 2019). The results of this study, extending insights from prior research (Huntjens et al., 2024b) that highlighted DBT’s significant impact on comorbid symptoms such as depression, further support the concept of DBT as a transdiagnostic intervention. This approach not only addresses distinct behaviours and symptoms, but also seems to target underlying mechanisms (Barlow et al., 2004; Tan et al., 2022).

Variability in core autism traits is characterised by inter-individual differences and intra-individual changes over time, spanning specific symptomatic domains such as social communication and restricted/repetitive behaviours (Waizbard-Bartov & Miller, 2023). This variability can be ascribed to distinct personal traits such as gender, IQ and sociodemographic factors and may be modulated by evolving developmental trajectories. Notably, behavioural interventions correlate with reduced restricted/repetitive behaviours and enhanced social skills, highlighting autism’s dynamic nature (Anderson et al., 2014; Cassidy et al., 2022; Costa et al., 2020; Elias & Lord, 2022; Hudry et al., 2018; Orinstein et al., 2014; Waizbard-Bartov & Miller, 2023).

### COVID-19 impact

The impact of the COVID-19 pandemic on autistic adults was profound, with escalating rates of infection and hospitalisation, increased distress and depression and diminished quality of life (Scheeren et al., 2023). Nonetheless, our clinical observations suggest that many autistic individuals found the reduced public social interactions and increased online engagement provided by pandemic restrictions beneficial. These individuals noted improvements such as reduced external stressors, increased autonomy over sensory exposures and a newfound sense of solidarity with their peers in coping with these shared challenges. This adaptive response may have enhanced their quality of life and social interactions. It might also account for our minimal attrition rate and explain why the COVID-adjusted analyses yielded results closely mirroring those from the base case analysis.

### Strengths and limitations

This study has several notable strengths, including its controlled design and recruitment from routine clinical settings, the inclusion of autistic individuals with significant levels of comorbidity, and the application of widely used DBT treatment protocols. These strengths increase the generalisability of the observed effects to patients in routine clinical practice and lend credibility to the feasibility of offering DBT to autistic individuals with suicidal behaviour in a clinical setting.

The study has several limitations. First, the reliance on self-reported outcomes risks socially desirable responses, though the scales demonstrated high test–retest reliability across clinical populations. Second, the psychological nature of the intervention prevented therapist and patient blinding, but outcome assessors remained blind to randomisation status. Third, while an 8.9% loss-to-follow-up occurred, a sensitivity analysis using the conservative LOCF method revealed outcomes comparable to those of the base case analysis. Fourth, COVID-19 lockdowns shifted our approach from in-person to virtual, but a sensitivity analysis confirms this change did not have a major impact on treatment outcomes. Fifth, the use of measures not psychometrically validated in autistic samples or created for the autistic population may affect the accuracy of the results. Sixth, DBT was offered in 6 months (not in the usual 12 months), and this intensity may have contributed to DBT’s effectiveness. Finally, there was limited training for clinicians new to DBT and low-to-moderate DBT adherence was observed.

### Implications for clinical practice

DBT was reported to be effective in reducing suicidal ideation, suicide attempts and depression. This article underscores the modest effects of DBT on social functioning and quality of life in autistic individuals. This promising evidence encourages clinicians and researchers to use DBT in clinical services and research.

### Implications for future research

Future studies should explore the potential benefits of incorporating booster sessions to better evaluate the enduring effects of DBT interventions on autistic individuals’ social outcomes. In addition, future research should use longer follow-up periods to adequately evaluate the regaining social roles in broader society.

## Conclusion

To our knowledge, this is the first RCT examining the effects of DBT treatment in autistic individuals, with a focus on clinically relevant secondary outcomes. DBT treatment significantly improved quality of life and social functioning, though modestly. Autistic traits showed no differences.

## Supplemental Material

sj-docx-1-aut-10.1177_13623613241302875 – Supplemental material for Secondary effects of dialectical behaviour therapy on social functioning, quality of life, and autism traits in autistic adults with suicidality Supplemental material, sj-docx-1-aut-10.1177_13623613241302875 for Secondary effects of dialectical behaviour therapy on social functioning, quality of life, and autism traits in autistic adults with suicidality by Anne Huntjens, LMC (Wies) van den Bosch, Bram Sizoo, Ad Kerkhof, Filip Smit and Mark van der Gaag in Autism
