# Supplementary material for: Secondary effects of dialectical behaviour therapy on social functioning, quality of life, and autism traits in autistic adults with suicidality
Source: Autism. 2024 Dec 14;29(5):1333–45. doi: 10.1177/13623613241302875 (PMC12038065; doi:10.1177/13623613241302875)
Supplement: sj-docx-1-aut-10.1177_13623613241302875 – Supplemental material for Secondary effects of dialectical behaviour therapy on social functioning, quality of life, and autism traits in autistic adults with suicidality [file sj-docx-1-aut-10.1177_13623613241302875.docx]

**Supplemental Material**

**Table 1.**Scoring Overview of Personal and Social Performance Scale (PSP)

|  | ***Difficulties in Domains of Functioning*** | | | |
| --- | --- | --- | --- | --- |
| ***Total Decile Score*** | ***1-Socially useful activities***  ***(e.g., work or study)*** | ***2-Personal and social***  ***relationships*** | ***3-Self-care*** | ***4-Disturbing and***  ***aggressive behaviors*** |
| *1-10  (death risk)* | Very severe | Very severe | Very severe | Very severe |
| *6-10 (no death risk)* | Very severe | Very severe | Very severe |  |
| *11-20 Option 1* | Severe | Severe | Severe | Severe |
| *Option 2* |  |  |  | Very severe |
| *21-30  Option 1* | Severe in two of these | | |  |
| *Option 2* |  |  |  | Severe |
| *31-40  Option 1* | Severe at least one & Marked at least one of these | | |  |
| *Option 2* |  |  |  | Marked |
| *41-50  Option 1* | Marked in at least two of these | | | |
| *Option 2* | Severe in one of these | | |  |
| *51-60  Option 1* | Marked in one of these areas | | |  |
| *Option 2* |  |  |  | Manifest |
| *61-70  Option 1* | Manifest in at least one of these | | |  |
| *Option 2* |  |  |  | Mild |
| *71-80* | Mild in at least one of these | | |  |
| *81-90* | Absent | Absent | Absent | Absent |
| *91-100* | Absent | Absent | Absent | Absent |

1 - PSP, Socially useful activities: Doing routine housework and carrying out household activities. Holding competitive or sheltered job. Attending school; 2=PSP, Personal and social relationships: Frequency of external social relations (e.g., meeting friends, going to appointments such as visits to the hairdresser). Participation in family life or life of the psychiatric centre;3=PSP, Self-care; bathing, brushing teeth, care of clothes making bed; 4 - PSP, Disturbing and aggressive behaviours: Rudeness, unsociability or whining; Speaking too loudly or speaking to others in a too-familiar manner. Eating in a socially unacceptable manner; Insulting others in public; Breaking or wreaking objects; Acting inappropriately (e.g., stripping or urinating in public); Quarreling or verbal threats; Physical assaults (Rabinowitz et al., 2021).

**Reference**

Rabinowitz, J., Opler, M., Rabinowitz, A. A., Negash, S., Anderson, A., Fu, D. J., Williamson, D., Kott, A., Davis, L. L., & Schooler, N. R. (2021). Consistency checks to improve measurement with the Personal and Social Performance Scale (PSP). *Schizophr Res*, *228*, 529–533. https://doi.org/10.1016/j.schres.2020.11.040
